# Adaptive multi-channel dehazing for enhanced visibility in underground coal mine images

**DOI:** 10.1371/journal.pone.0334251

**Published:** 2025-11-05

**Authors:** Yingbo Fan, Shanjun Mao, Mei Li, Boxiang Yang, Yinglu Yang

**Affiliations:** 1 Institute of Remote Sensing and Geographic Information Systems, Peking University, Beijing, China; 2 China University of Mining & Technology, Beijing, China; Hunan Normal University, CHINA

## Abstract

Image dehazing has gained significant attention due to its importance in enhancing image clarity in various applications. However, existing algorithms often struggle with suboptimal performance in underground coal mine environments, characterized by dim lighting and atmospheric interference. This paper presents an adaptive multi-channel dehazing algorithm tailored for enhancing images from underground coal mines. By utilizing an improved color attenuation prior method, the algorithm effectively detects fog density, incorporating texture information and illumination invariance features from the HSV space for enhanced adaptability and robustness. The algorithm segregates foggy and fog-free image regions, applying image enhancement in clear areas and threshold multi-channel inspection dehazing in foggy regions. A multi-scale pyramid and guided filtering approach are employed to refine the estimation of image transmittance, mitigating blocky artifacts. For video dehazing, a parameter reuse mechanism leveraging inter-frame similarity significantly improves real-time performance. Experimental results on coal mine datasets and public benchmarks demonstrate that the proposed algorithm outperforms existing methods in defogging effectiveness, computational efficiency, and stability, rendering it suitable for real-time applications such as safety monitoring in underground coal mines.

## Introduction

As security surveillance becomes more widespread and computer vision rapidly advances, the demand for acquiring clear, high-quality images is increasing [1,2]. Consequently, in the current field of digital image processing, image dehazing has gained extensive research attention as a critical method for enhancing image clarity [3,4]. Because atmospheric disturbances, such as haze, smoke, and dust, are prevalent in most scenes and often severely affect image quality and visual performance, they can result in loss of image details, color distortion, and reduced contrast [5,6].

To overcome the adverse effects of atmospheric interference on image quality, numerous advanced dehazing techniques based on traditional methods and deep learning have emerged. These techniques aim to restore clear visual information from haze-affected images, making them closer to real-world scenes [7]. However, in practical applications, current image dehazing algorithms still face several challenges and issues [8,9]. Firstly, traditional dehazing methods based on physical models often rely on accurate estimation of environmental lighting and atmospheric scattering models. These parameters are usually difficult to obtain directly, resulting in insufficient stability and robustness of the algorithms [10,11]. Secondly, although deep learning-based image dehazing algorithms have achieved remarkable results, their performance in handling complex scenes and varying weather conditions remains suboptimal [12,13]. Deep neural networks typically require a large amount of labeled data for training, and obtaining large-scale labeled data in real-world scenarios poses significant difficulties and challenges [14]. Furthermore, there is often a trade-off between detail restoration and maintaining vivid color in image dehazing algorithms [15]. Most current algorithms struggle to simultaneously achieve both high detail restoration and enhanced color fidelity in the processed images.

In production environments like coal mines, which are typically dimly lit, the quality of underground images is often affected by atmospheric interferences such as haze and coal dust [16]. These factors lead to significant blurring and degradation of images, severely impacting their visibility and the ability to extract information [17]. Therefore, the research and application of image dehazing technology are crucial for enhancing underground monitoring and ensuring safe production in coal mines [18,19]. However, most current image dehazing algorithms are only suitable for specific types of haze or atmospheric interference, and their effectiveness in the complex and variable conditions of underground coal mines is not guaranteed [20]. Additionally, existing algorithms often require substantial computational resources, resulting in long processing times which are not conducive to real-time monitoring and emergency response. Moreover, some algorithms may introduce distortions or artifacts during the dehazing process, compromising the accuracy and reliability of the images [21]. These issues present significant challenges to the safety monitoring and production efficiency in coal mining environments [22].

**Fig 1:**
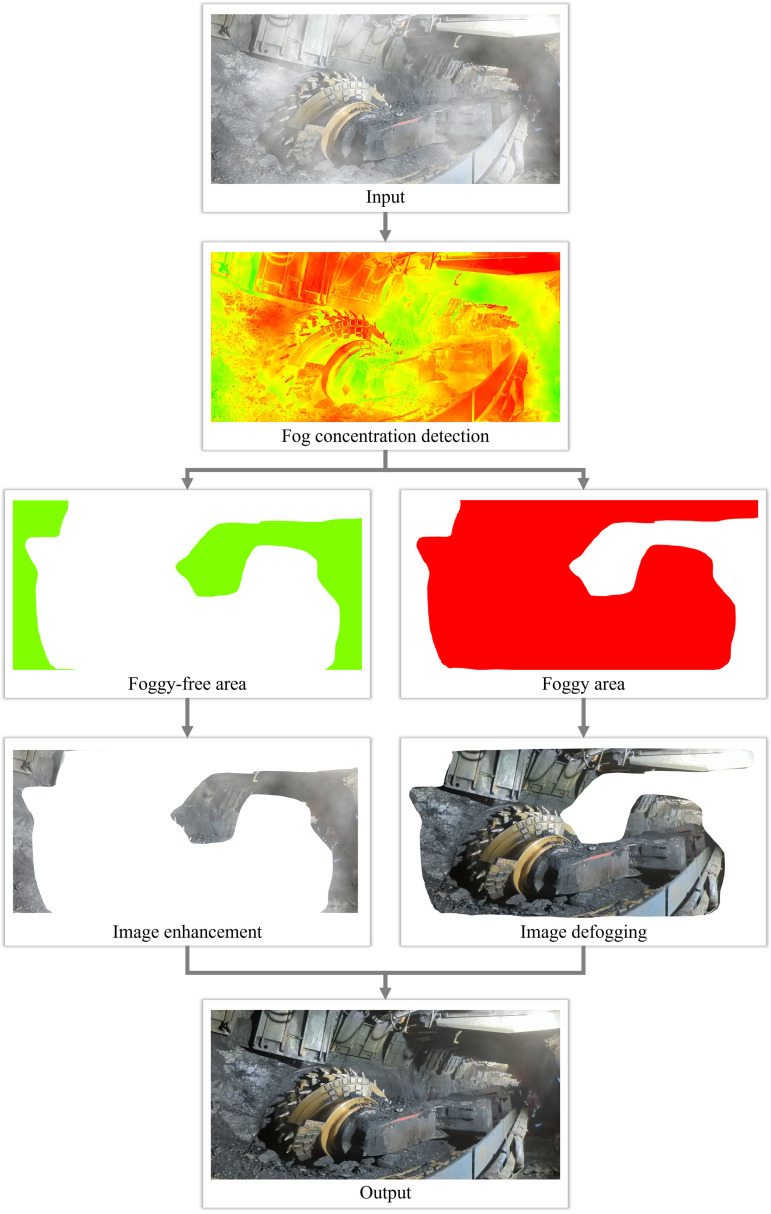
Flowchart of image defogging algorithm based on thresholding multi-channel inspection.

To better address the urgent demand for image dehazing in industrial production environments such as coal mines, this paper proposes an adaptive multi-channel dehazing for enhanced visibility in underground coal mine images (AMCD). The algorithm first detects haze concentration using an improved color attenuation prior method. It then enhances the image in haze-free regions and performs threshold multi-channel verification dehazing in hazy regions, ultimately outputting a complete dehazed image. The brief process is illustrated in Fig 1. The proposed algorithm is designed to be adaptive and robust in complex industrial production environments. It ensures the accuracy and reliability of the dehazing effect, meeting the practical needs of safety monitoring and production management in environments with uneven lighting, such as coal mines. This approach provides a reference for the further development of image dehazing technology.

## Related work

Currently, there is a significant amount of research focused on image dehazing, which can be primarily categorized into two types: prior-based dehazing algorithms and deep learning-based dehazing algorithms [23]. Prior-based image dehazing algorithms can be further divided into image enhancement-based and image restoration-based dehazing algorithms, based on different principles. Image enhancement-based dehazing algorithms typically aim to restore clear, haze-free images by removing noise and improving image contrast. These methods focus on enhancing the visual quality of the image without necessarily modeling the physical properties of the haze [24].

For example, Chen proposed a single-image dehazing method based on adaptive histogram equalization and gamma correction linearization [25]. This method is extremely fast and suitable for video sequence data. Li proposed a method that utilizes Retinex theory and Taylor series expansion to denoise nighttime images, referred to as “RDT”. During the image fusion and color conversion process, atmospheric light images and potential haze-free images are used to obtain the final haze-free image [26]. Khan calculated atmospheric light for a given hazy image by performing wavelet domain decomposition and retaining high-frequency sub-bands [27]. Dense haze removal is then carried out on the approximate low-frequency sub-bands of the given hazy image. Image restoration-based methods mainly include the dark channel prior algorithm proposed by He, which restores clear images by inversely solving the formation process of hazy images, laying the foundation for subsequent methods [28]. For instance, Li proposed a method to estimate the parameters in the physical model based on an improved bright channel prior and dark channel prior [29]. This method effectively addresses the issue where the dark channel prior fails in sky regions. Generally, dehazing effects based on the atmospheric degradation model are superior to those of image enhancement-based dehazing algorithms [30].

Advantages of deep learning neural networks in image task processing have also led to a significant number of neural network-based approaches in dehazing algorithms. The first category of methods is based on the atmospheric degradation model, utilizing neural networks to estimate parameters within the model [31]. For instance, Cai proposed a trainable end-to-end system (DehazeNet) for estimating the transmission rate [32]. DehazeNet takes a hazy image as input and outputs the transmission rate, which is then used to invert the atmospheric scattering model to recover a haze-free image. Chi Yoon designed a scaled convolution group to handle computationally intensive operations at a low resolution, thereby reducing the dehazing processing time of the end-to-end network model without compromising performance [33]. The second category of CNN methods utilizes the input hazy image to directly output a dehazed image. For example, Li proposed an All-in-One Dehazing Network (AOD-Net), which generates a clear image directly through a lightweight CNN [34]. This novel end-to-end design facilitates the integration of AOD-Net into other deep models (e.g., faster R-CNN) to enhance the performance of high-level tasks on blurry images. Manu suggested using a Generative Adversarial Network (GAN) for dehazing given blurry input images [35]. The proposed GAN architecture employs a Feature Residual Dense Network (FRDN) as the generator and a Markov discriminator with additional layers (PatchGAN) as the discriminator. This approach enhances the visibility of the scene and the authenticity of the dehazed images.

## Method

### Fog concentration detection based on an improved color decay prior

Traditional color attenuation prior suggests that the luminance and saturation values of a hazy image significantly change with the increase of fog density, which is used for fog density detection and removal [36]. However, this method heavily relies on the color attenuation features of the scene, which may lead to poor performance under different scenes or lighting conditions [37]. Moreover, some scenes may have complex color distributions, and traditional algorithms may not effectively handle this diversity, leading to increased errors [38]. Therefore, this paper proposes an improved method for fog density detection based on the color attenuation prior. The algorithm introduces texture information of the image in the HSV space to enhance the adaptability of the algorithm to different scenes. Additionally, the introduction of illumination invariance features makes the algorithm more robust to changes in lighting.

**Fig 2:**
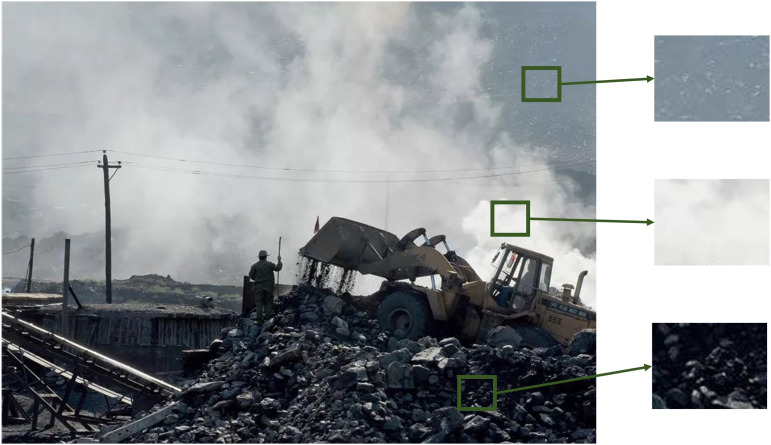
Images with different fog areas.

**Fig 3:**
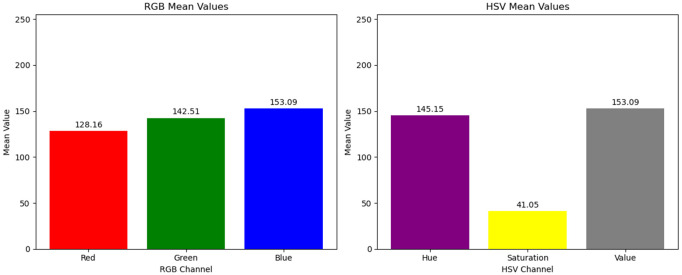
RGB and HSV differences of pixel blocks under different fog concentrations (heavy fog).

**Fig 4:**
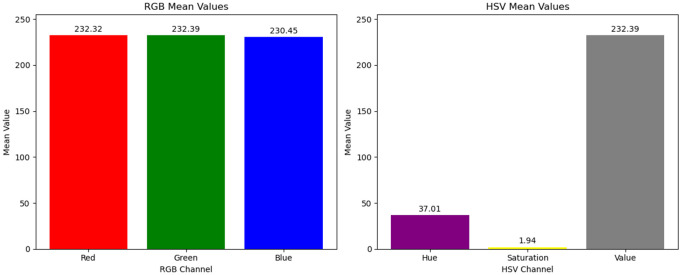
RGB and HSV differences of pixel blocks under different fog concentrations (moderately dense fog).

**Fig 5:**
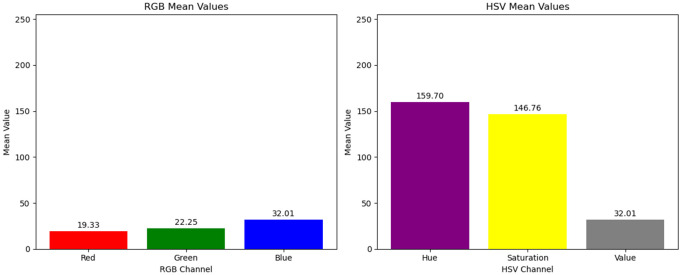
RGB and HSV differences of pixel blocks under different fog concentrations (light fog).

Fig 2 illustrates the comparison and differences in RGB and HSV color values for pixel blocks with varying fog concentrations in a dimly lit scene throughout the day. As shown in Fig 3, for fog-free areas, the luminance (V channel) and saturation (S channel) exhibit relatively higher average values (V: 120–150, S: 80–110), with minimal differences between them (<10). As fog density increases (Fig 4 and Fig 5), luminance values further increase (V: 160–220), while saturation sharply decreases (S: 20–60), causing larger differences (>100) between luminance and saturation. This paper extracts frames from video within a coal mine scene, obtaining 20,000 foggy images under various production scenarios. Based on these images, the aforementioned test was validated, and the conclusion confirms that in dimly lit scenes like coal mines, the RGB and HSV color values generally conform to the observed patterns.

Based on this prior knowledge, this paper proposes an improved fog density detection method that takes into account color attenuation, converting images from the RGB color space to the HSV space, and calculating the fog density using both color spaces. The image is divided into multiple pixel blocks of a certain size, and for each pixel block B, the initial fog concentration in the HSV space is calculated using Equation (1) as follows:


$${f_{hsv}\left(x\right)|}_{x\in B}=\frac{\text{1}}{N}\sum_{x=1}^{N}\frac{v(x)-s(x)}{h(x)+s(x)+v(x)}$$ (1)

The formula *f_hsv_(x)* represents the initial fog density for the current pixel block in the HSV space, where *N* is the total number of pixels within block *B*, and *h(x)*, *s(x)*, and *v(x)* are the channels of the image in the HSV space. The initial fog density in the RGB space is calculated as shown in Equation (2):


$${f_{rgb}\left(x\right)|}_{x\in B}=\frac{\text{1}}{N}\sum_{x=1}^{N}r(x)+g(x)+b(x)$$ (2)

The formula *f*_*rgb*_*(x)* represents the initial fog density for the current pixel block in the RGB space, with *r(x)*, *g(x)*, and *b(x)* being the channels of the image in the RGB space. Finally, the maximum values of *f*_*hsv*_*(x)* and *f*_*rgb*_*(x)* are normalized and then added together to obtain the final estimate of the initial fog density, as shown in the following Equation (3):


$${f(x)=f}_{hsv}^{n}{(x)+f}_{rgb}^{n}\left(x\right)$$ (3)

After obtaining the initial fog density estimate, an adaptive threshold is used to perform a binarization segmentation operation on the fog density. The connected region with the maximum fog content is extracted, and the area of this region is calculated. The area of the fog-containing connected region is compared with the area threshold. If it is greater than the area threshold, the image is classified as foggy; otherwise, it is classified as non-foggy.

### De-fogging algorithm based on multi-channel threshold inspection

In computer vision, the model is usually defined as Equation (4) for haze images:


I(x)=J(x)t(x)+A(1−t(x)) (4)

In the model, *I(x)* represents the foggy image, *J(x)* represents the clear image without fog, *t(x)* denotes the transmission rate, which primarily describes the portion of light that is not scattered as it passes through the medium and reaches the camera [39]. A represents the global atmospheric light. The first term of the formula is called the direct attenuation term, which describes the attenuation of light as it propagates through the medium in the scene captured by the image. The second term is called the atmospheric light, which is mainly caused by scattering in front of the image acquisition device and can easily lead to a shift in the scene’s hue [40]. When the atmosphere is uniformly distributed in the scene, the transmission rate *t(x)* can be expressed as shown in Equation (5):


$$t(x)=e^{-\beta d(x)}$$ (5)

In Equation (6), β represents the scattering coefficient of the atmosphere. This formula indicates that as the depth of the scene increases, the brightness of the scene decreases exponentially. Therefore, if the transmission rate can be estimated with reasonable accuracy, it is possible to recover the clear image without fog effectively [41].

#### Scene atmospheric light value estimation.

As stated by the atmospheric scattering model, accurately estimating the global atmospheric light value is crucial for the subsequent accurate estimation of the transmission rate [42]. Furthermore, the accuracy of the transmission rate estimation directly impacts the subsequent image dehazing process. The atmospheric scattering model explains that as the fog concentration increases, the scattering effect of the reflected light from scene objects intensifies, leading to brighter colors in the image scene [43]. Therefore, a common practice is to consider the brightest color in the image as the global atmospheric light value. However, in certain scenes, objects may exhibit brightness levels that exceed the atmospheric light value, such as light sources in environments like underground mines [22]. Failing to account for this scenario may lead to incorrect estimations, which in turn affects the accuracy of the transmission rate estimation. Therefore, it is necessary to adopt methods that can correctly identify and handle such special cases to ensure the accuracy of the global atmospheric light value estimation, and subsequently, the accuracy of the transmission rate estimation [44].

Therefore, this paper adopts the approach of constructing a multiscale pyramid followed by using a threshold multi-channel prior method to estimate the local minimum atmospheric light value. Specifically, the first step is to construct the multiscale pyramid of the image. This can be achieved by downsampling the original image *I(x)* at various levels to obtain a sequence of images with different resolutions, denoted as *I*_*s*_*(x)*^*S*^_*s=1*_, where *S* represents the number of scales. Given that the overall illumination in a coal mine environment is dark and the light distribution is extremely uneven, using the traditional dark channel check cannot accurately estimate the atmospheric light value within the scene. Hence, the threshold multi-channel prior method is employed to calculate the corresponding image *J*_*s*_*(x)* for each scale *s* is computed as shown in Equation (6):


$$J_{s}(x)=\underset{y\in B(x)}{min}(\omega_{r}I_{s,r}(y)+\omega_{g}I_{s,g}(y)+\omega_{b}I_{s,b}\left(y)\right)$$ (6)

Where *I*_*s,r*_*(y)* denotes the pixel value of image *I*_*s*_*(x)* on channel *r*, *I*_*s,g*_*(y)* denotes the pixel value of image *I*_*s*_*(x)* on channel *g*, *I*_*s,b*_*(y)* denotes the pixel value of image *I*_*s*_*(x)* on channel *b*, *B(x)* denotes a region of a block of pixels centered on pixel *x*, and w_r_, w_g_, and w_b_ denote the proportion of weighting values of the individual channels of the RGB, respectively.

The local minimum atmospheric light values estimated on different scales are integrated by weighted averaging, where different image resolutions correspond to different weight values and higher resolution images are given higher weights. Finally, the local minimum atmospheric light values estimated at different scales are weighted and averaged together to obtain the final estimated global atmospheric light value *A*. The final estimated global atmospheric light value *A* is obtained by integrating the local minimum atmospheric light values estimated at different scales with the weighted average as shown in Equation (7):


$$A=\;\frac{\sum_{s=1}^{S}\omega_{s}\cdot A_{s}}{\sum_{s=1}^{S}\omega_{s}}$$ (7)

The w_r_ in Equation (7). denotes the extent to which the corresponding image resolution of each scale contributes to the integration results.

#### Image transmittance estimation.

Assuming that the scene depth is the same within a pixel block *B*, the transmission rate *t(x)* is the same for all pixels in block *B*. For each pixel block, its contrast is as shown in the Equation (8):


$$C_{MSE}=\sum {\mathrm{\backslash nolimits}}_{c\in \{r,g,b\}}\sum_{x=1}^{N}\frac{{\left(J_{c}(x)-{\bar{J}}_{c}\right)}^{2}}{N}$$ (8)

In the Equation (8), J_*c*_ is a single-channel fog-free image and $\overset{―}{J_{c}}$ is the mean value of *J*_*c*_ *(x)* within the pixel block *D*. It can be obtained by bringing Equation (1) into the above equation:


$$C_{MSE}=\sum {\mathrm{\backslash nolimits}}_{c\in \{r,g,b\}}\sum_{x=1}^{N}\frac{{\left(I_{c}(x)-{\bar{I}}_{c}\right)}^{2}}{t^{2}N}$$ (9)

In Equation (9), I_*c*_*(x)* is a single-channel fogged image, and $\overset{―}{I_{c}}$ is the mean value of *I*_*c*_*(x)* within the pixel block D. As indicated by Equation (1), there exists a linear relationship between the output value *J(x)* and the input value *I(x)*. When the input value falls within an intermediate range, the mapped output value will reside within the interval [0, 255]. The input value is directly dependent on the transmittance value *t*. For input values outside this range, the output values will be truncated, leading to a loss of color information in the image [45]. *C*_*MSE*_ is a monotonically decreasing function of the transmittance *t*; a transmittance *t* that is too small will result in excessive truncation of the recovered pixel values, causing severe loss of color information. Therefore, while maximizing contrast, it is essential to minimize the loss of color information to the greatest extent possible. According to the constraint condition that minimizes color loss, the transmittance t should satisfy the following condition formula:


$$t\geq \max\{\begin{array}{c} {}*20c\underset{y\in B(x)}{min}\frac{((\omega_{r}I_{s,r}(y)+\omega_{g}I_{s,g}(y)+\omega_{b}I_{s,b}(y))-A_{c})}{-A_{c}}\\ \underset{y\in B(x)}{max}\frac{((\omega_{r}I_{s,r}(y)+\omega_{g}I_{s,g}(y)+\omega_{b}I_{s,b}(y))-A_{c})}{\text{255}-A_{c}} \end{array}$$ (10)

And the contrast cost function can be expressed by *E*_*C*_ with the formula shown below Equation (11):


$$E_{C}=-C_{MSE}=\sum {\mathrm{\backslash nolimits}}_{c\in \{r,g,b\}}\sum_{x=1}^{N}\frac{{\left(I_{c}(x)-{\bar{I}}_{c}\right)}^{2}}{t^{2}N}$$ (11)

Eventually the problem of transmittance estimation is converted to solving for the minimum of the cost function E, as shown in Equation (12):


$$E=E_{C}+E_{I}$$ (12)

In practical applications within industrial scenarios, such as coal mines, one of the challenges in the image restoration and defogging task is to handle the complex scenes present in the image. In these scenes, each pixel block may correspond to multiple different scene targets, leading to variations in their scene depth and transmittance [46]. In such cases, traditional pixel block-based transmittance estimation methods may result in noticeable blockiness after image recovery, which diminishes the visual quality and realism of the image.

To overcome this issue, a guided filtering approach can be employed to transform the transmittance estimation from a pixel block-based method to a pixel-based method, thereby alleviating or eliminating the occurrence of blockiness while recovering image details. This method can smooth the transmittance within local regions based on the structure and features of the image. Unlike traditional mean or Gaussian filtering, guided filtering takes into account the similarity and correlation between pixels in the image, better preserving the detailed information. Moreover, guided filtering elevates the estimation of transmittance from the pixel block level to the pixel level. By estimating the transmittance within a local window surrounding each pixel point in the image, rather than considering only the average transmittance of the entire pixel block, the transmittance can more finely reflect the variations in optical properties across different regions of the image. The guided filtering process applied to the image I(x) can be represented by the following Equation (13):


$$\bar{t}(x)=S^{T}I(x)+b$$ (13)

In the equation, *t(x)* represents the filtered transmittance; *S* is the scaling coefficient; and *b* is the bias value. By using the conjugate gradient method to iteratively minimize the error between the initial transmittance and the filtered transmittance, the optimal solutions for the scaling coefficient and bias can be obtained.

#### Time continuity factor.

**Fig 6:**
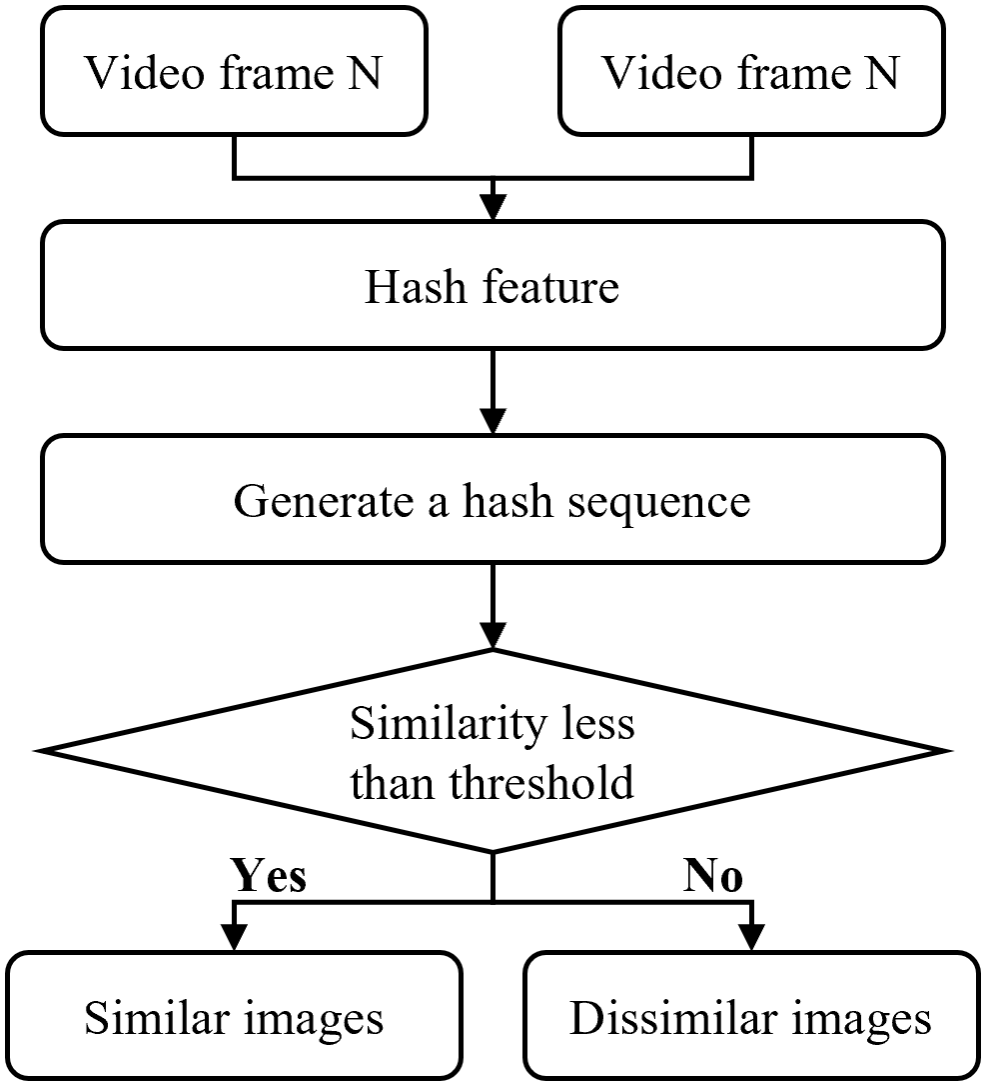
Schematic diagram of the principle of perceptual hashing.

Considering the continuity of video frames, this paper proposes a method to determine whether recalculation of parameters such as transmittance is necessary by detecting the similarity between video frames, thereby reducing redundant computations and enhancing the real-time performance of video dehazing. The perceptual hashing method can be employed to compare the similarity between the current frame and the previous one, determining whether the current dehazing parameters can be retained. Furthermore, the perceptual hashing method converts images or video frames into grayscale to simplify computational load and accelerate algorithm execution [47]. The implementation principle of the perceptual hashing algorithm is illustrated in the Fig 6.

The perceptual hashing algorithm, when assessing the similarity between the original image and the image under test, first extracts the hash features of both images. After obtaining the unique hash feature values for each image, they are compared [48]. If the Hamming distance between the hash feature sequences of the original frame and the next frame is less than a predetermined threshold, the two images are deemed similar. Conversely, if the Hamming distance exceeds the threshold, the images are considered dissimilar. Since the hash features of an image are unique, it ensures that identical images will not yield different feature sequences, thereby offering a high level of accuracy. The selection of the distance threshold is determined empirically through experiments, and it is used to judge the similarity of images based on this threshold.

To enhance image details and improve the accuracy of the algorithm’s detection, this paper utilizes the Discrete Cosine Transform (DCT) to convert the images undergoing similarity detection into the frequency domain. Subsequently, appropriate elements from the obtained frequency coefficient matrix are selected to calculate the image hash feature sequence. The formula for the Discrete Cosine Transform (DCT) is expressed as Equation (14):


$$\left\{ \begin{array}{c} F(0,0)=\frac{1}{k}\sum_{i=0}^{k-1}\sum_{j=0}^{k-1}f(i,j)\\ F(0,y)=\frac{\sqrt{2}}{k}\sum_{i=0}^{k-1}\sum_{j=0}^{k-1}f(i,j)⬝\cos\frac{(2j+1)y\pi }{2k}\\ F(x,0)=\frac{\sqrt{2}}{k}\sum_{i=0}^{k-1}\sum_{j=0}^{k-1}f(i,j)⬝\cos\frac{(2i+1)x\pi }{2k}\\ F(x,y)=\frac{2}{k}\sum_{i=0}^{k-1}\sum_{j=0}^{k-1}f(i,j)⬝\cos\frac{(2i+1)x\pi }{2k}⬝\frac{(2j+1)y\pi }{2k} \end{array}\right.$$ (14)

In the equation, *i* and *j* represent the coordinates of elements in the pixel domain of the image, with *f(i, j)* being the corresponding value of the element. The variable *n* denotes the order of the pixel matrix. The variables *x* and *y* represent the coordinates of elements in the frequency domain of the image, with *F(x, y)* being the elements of the coefficient matrix in the frequency domain after transformation. The coefficient matrix is denoted as $N_{k\times k}$, where $n_{m\times m}$ represents the top-left m×m matrix. The steps of the perceptual hash algorithm are as shown in Algorithm 1.

- Step 1: Initially, transform two adjacent frames of the image into a *k × k* matrix, resulting in a total of $k^{2}$ pixels.
- Step 2: Convert the transformed image into a grayscale image.
- Step 3: Perform a DCT on the image, transitioning from the pixel domain to the frequency domain. Calculate the frequency coefficient matrix $N_{k\times k}$ and denote the top-left *m × m* matrix of this coefficient matrix as $n_{m\times m}$.
- Step 4: Compute the average value of the frequency coefficient matrix $n_{m\times m}$, denoted as $n_{avg}$.
- Step 5: Traverse each element $n_{i}$ in $n_{m\times m}$ and compare $n_{i}$ with $n_{avg}$. If $n_{i}$ is greater than $n_{avg}$, assign a value of 1; otherwise, assign a value of 0. This generates the perceptual hash value of the image.
- Step 6: After obtaining the hash feature sequences of the two images, compare the Hamming distance of the feature sequences with a predetermined threshold to determine the similarity of the images.

**Algorithm 1.** Sense Hash Algorithm

**Input** Two adjacent frames: *I_N_, I_N+1_*; threshold *θ*

**Output:** Similarity of two neighboring image frames

1: Transform two neighboring frames to *k × k* for a total of *k*^2^ pixels

2: Converting the transformed image to a grayscale map

3: Discrete cosine transform applied to the image to obtain the frequency coefficient matrix *N*_*k×k*_ and the *m × m* matrix in the upper left corner of the matrix is denoted as *n*_*m×m*_

4: Calculate the average of the frequency coefficient matrix *n*_*m×m*_, denoted as *n*_*avg*_

5: **for** *n*_*i*_ in *n*_*m×m*_ **do**:

6:    **if** *n*_*i *_*>* *n*_*avg*_ **do:**

7:       Denoted as 1

8:    **else do:**

9:       Denoted as 0

10: Get the hash feature sequence of the two images *H_N_, H_N+1_*

11: **if** d (*H_N_, H_N+1_*)> *θ* **do:**

12:    Two adjacent images are not similar

13: **else do:**

14:    Two adjacent images are similar

## Experiments

The image dehazing experiments in this paper were conducted on a workstation with the following specifications: CPU: Intel Core i9-13900HX (2.2 GHz, 6 cores, 12 threads), RAM: 16GB DDR4 2400MHz, GPU: NVIDIA GeForce GTX 4070 Ti (8GB GDDR5X). The algorithm was implemented using Python 3.6 and OpenCV 3.4.3. Due to the limited availability of public datasets for image dehazing research in dark environments and other industrial production settings, this study, in addition to validation on a self-collected dataset of coal mine faces with and without fog, also conducted validations on publicly available datasets NH-Haze2 [49], Dense-Haze [50], and SOTS-indoor [51]. The experimental parameters were set consistently with those described in C^2^PNet [52].

### Fog concentration detection segmentation effect

During the process of image dehazing, applying dehazing algorithms to clear, fog-free images can not only lead to inefficient use of computational resources but can also backfire, potentially causing problems such as overexposure or uneven contrast which further degrade image quality [53]. To allocate computational resources more effectively, this paper proposes a fog density detection method based on an improved color attenuation prior. This method can detect the presence of fog in an image and decide whether to apply the subsequent dehazing algorithm based on the detection result. To validate the effectiveness of this approach, images of coal mining machines at the darker coal mine faces were selected as test samples. In the experiments, a fog area threshold of 25% was set, meaning that if the area of fog pixels in an image exceeds 25% of the total number of pixels, the image is determined to be foggy.

**Fig 7:**
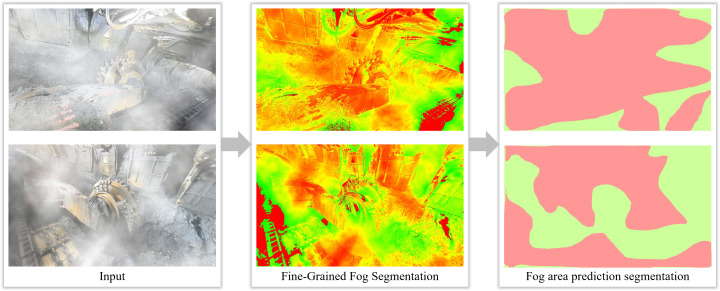
Schematic diagram of the effect of coal mine belt fog concentration detection and area segmentation of fogged images.

**Fig 8:**
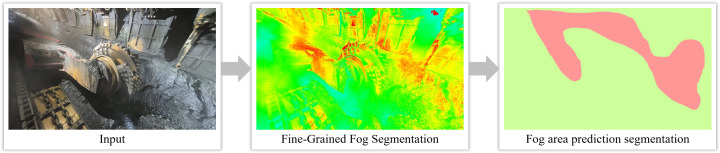
Schematic diagram of the effect of coal mine belt fog concentration detection and area segmentation of non-fogged images.

The experimental results, as shown in Fig 7 and Fig 8, demonstrate that the fog density detection method based on the improved color attenuation prior can accurately identify the distribution of fog in images. It is capable of identifying the largest connected foggy region in the fog concentration distribution map, where red areas represent foggy regions and green areas represent non-foggy regions. For instance, in the first set of images in Fig 7, the detected largest connected foggy region accounts for 76.35% of the total pixel count, which exceeds the set threshold of 25%. Therefore, this image is classified as foggy and requires dehazing treatment. Conversely, in the image of Fig 8, the largest connected foggy region constitutes only 16.87% of the total pixels, which is below the threshold, thus classifying the image as non-foggy and exempting it from dehazing operations. This fog density detection method intelligently identifies foggy images and automatically skips non-foggy ones, thereby targeting dehazing operations only where necessary, enhancing processing efficiency and the utilization of computational resources. Additionally, prior detection of foggy regions in images can optimize the video image processing workflow, especially in resource-constrained environments like coal mine faces, where it holds significant practical importance.

### De-fogging effect based on thresholded multi-channel test

In order to evaluate the practical effectiveness of the threshold-based multi-channel inspection defogging method proposed in this study in foggy video scenes of underground coal mine working face, an exhaustive experimental comparative analysis is conducted in this paper. The image de-fogging algorithms such as AODNet [34], GDN [54], DeHamer [55] and C^2^PNet [52] are selected as the control group for comparative analysis. In terms of dataset this paper uses the image dataset constructed by extracting frames from some of the coal mine underground videos, and also adopts the coal mine underground dataset, public datasets such as NH-Haze2, Dense-Haze, and SOTS-indoor for comparative testing.

#### Qualitative comparison results.

**Fig 9:**
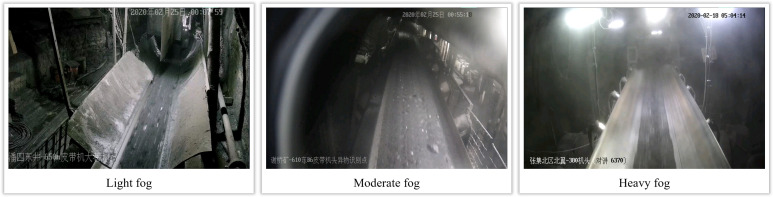
Schematic diagram of different fog concentrations in a coal mine.

In this study, qualitative experiments are designed to measure the performance of the de-fogging algorithm proposed in this paper in foggy videos from coal mine underground through intuitive visual evaluation. These experiments fully consider the subjective perception of the defogging effect and the adaptability in different scenes. In this paper, we first construct a dataset of underground coal mine images containing a variety of fog concentrations and scene complexities based on sampling frames from selected surveillance videos from a coal mine underground. These images were selected to cover a wide range of conditions from light to dense fog, as well as different lighting and environmental settings, thus ensuring the comprehensiveness of the test. One of the images of this coal mine dataset with different fog concentrations is shown in Fig 9.

**Fig 10:**
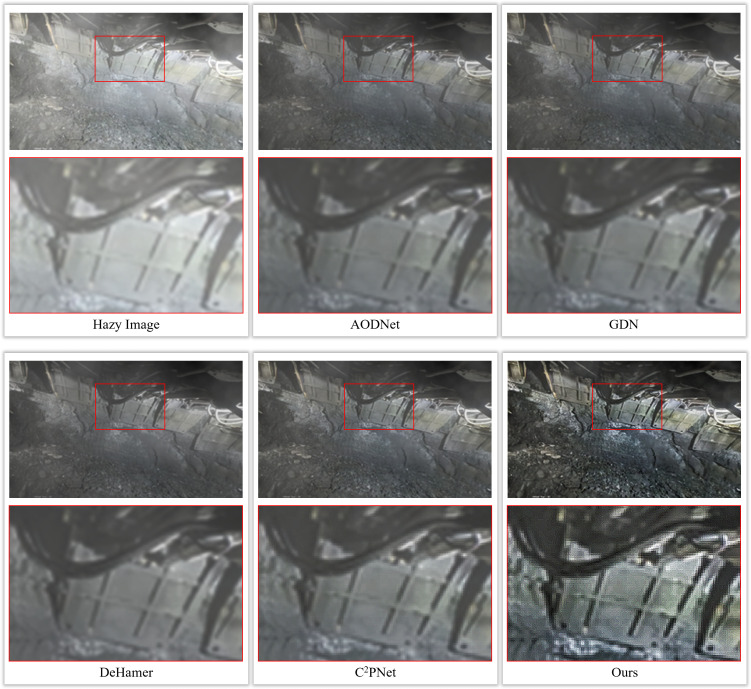
Comparison of coal mine gangplank image and other defogging algorithms’ defogging effect.

Threshold-based multi-channel test defogging method is applied to these images and compared with the intuitive visualization of multiple existing defogging algorithms as shown in Fig 10.

**Fig 11:**
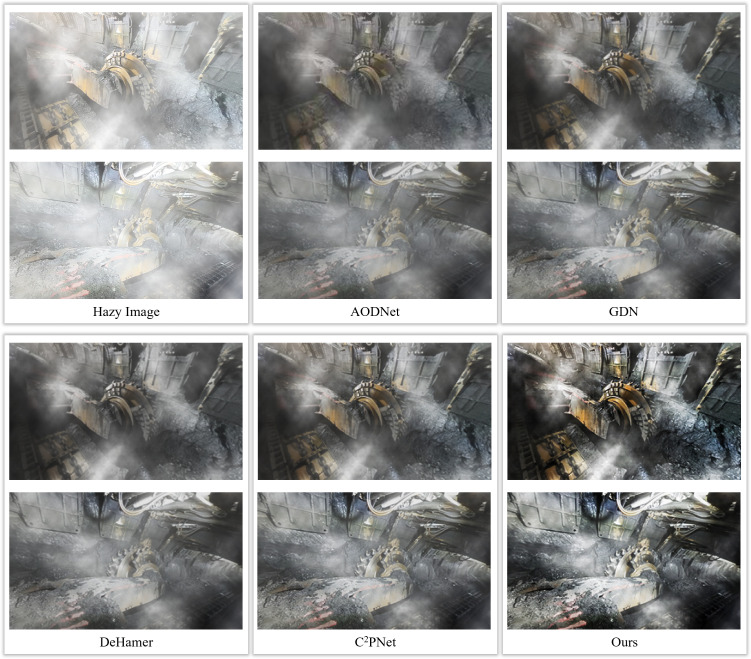
De-fogging effect of AMCD and other de-fogging algorithms on coal mine images with different concentrations.

As can be seen from the figure, compared with other defogging algorithms, the algorithm in this paper performs relatively well in terms of image clarity, completeness of detail retention, and restoration of color fidelity. Considering the diversity of working environments in underground coal mines, this paper tests the defogging algorithm in different underground scenarios (including different lighting conditions, various fog concentrations, etc.). The purpose of these tests is to evaluate the adaptability and robustness of the algorithm to different environmental conditions. When dealing with the complex scenes in the underground, the defogging algorithm in this paper also has good results in displaying the details of mechanical equipment, the degree of fog dissipation in the far and near areas, and the clarity of the edges of people and objects. In addition, this paper also observes the comprehensive visual effect of the image after defogging, including the improvement of contrast, saturation and brightness, as shown in Fig 11.

#### Quantitative comparison results.

Quantitative experimental comparisons of the defogging algorithms are a key part of this study used to quantitatively assess the performance of the defogging algorithms proposed in this paper. These experiments aim to accurately measure the defogging effect, computational efficiency, and stability of the algorithms through a series of objective metrics. In order to quantitatively assess the defogging effect, we use a variety of objective metrics, including peak signal-to-noise ratio (PSNR) and structural similarity index (SSIM). Together, these metrics assess the similarity and difference between the defogged image and the original fog-free image. Among them, PSNR mainly measures the quality of image reconstruction, with higher values indicating better image quality, while SSIM evaluates the structural similarity between the two images, with values close to 1 indicating that the defogged image is visually closer to the original. The comparisons of AMCD with other methods on the above metrics are shown in Table 1.

**Table 1:** Comparison of quantitative evaluation of different defogging algorithms.

Method	NH-Haze2		Dense-Haze		SOTS-indoor		Core mine		#Params
	PSNR	SSIM	PSNR	SSIM	PSNR	SSIM	PSNR	SSIM	
AODNet^34^	12.34	0.6312	12.82	0.4683	19.06	0.8504	11.64	0.4309	0.02M
FSAD-Net^56^	14.54	0.6250	13.06	0.5038	23.41	0.9336	11.93	0.4681	0.47M
GDN^54^	19.26	0.5326	14.96	0.5326	32.16	0.9836	13.82	0.5227	0.96M
DeHamer^55^	19.18	0.7939	16.62	0.5602	36.63	0.9881	15.93	0.5515	132.45M
C^2^PNet^52^	21.19	0.8334	16.88	0.5728	42.56	0.9954	16.09	0.5610	7.17M
Ours	21.85	0.8491	16.89	0.5740	44.34	0.9958	16.86	0.5703	0.002M

This paper compares the proposed dehazing algorithm with several existing algorithms, including traditional dehazing algorithms and the latest deep learning methods. It is evident that, under the premise of a very small number of parameters, the performance of the algorithm proposed in this paper surpasses that of traditional algorithms and some deep learning algorithms on most public datasets. By running all algorithms under the same hardware and software conditions, the fairness of the comparison is ensured. Comparative experiments not only assess the relative advantages of our algorithm in terms of dehazing effect but also reveal differences in processing speed and resource consumption among the various algorithms.

In addition to the quantitative assessment of dehazing effects, computational efficiency is another important aspect considered in this paper for evaluating dehazing algorithms. This paper records the average time required by the algorithm to process a single frame image, thereby assessing the algorithm’s real-time processing capability. This metric is particularly important for real-time video monitoring systems in coal mines, as high latency could affect the system’s responsiveness and practicality. Similarly, using a coal mine dataset as the test set, this paper tests the differences in dehazing effects and time consumption among different dehazing algorithms, as shown in Table 2.

**Table 2:** Comparison of quantitative evaluation of different defogging algorithms.

Method	MSE	Time (ms)
AODNet [34]	45.63	33
FSAD-Net [56]	23.59	184
GDN [54]	34.02	105
DeHamer [55]	21.95	224
C^2^PNet [52]	17.29	319
Ours	17.27	29

The results of the quantitative experiments provide a comprehensive set of data for the effects of various dehazing algorithms, enabling this paper to evaluate the performance of the proposed algorithm from multiple dimensions. By analyzing metrics such as PSNR and SSIM, this paper obtains an objective measure of the dehazing effect of the algorithm. Additionally, through computational efficiency analysis, this paper gains a deeper understanding of the feasibility of the threshold-based multi-channel inspection dehazing algorithm in practical applications. The comprehensive results of the quantitative experiments reveal that the proposed dehazing algorithm not only ensures high-quality dehazing effects but also demonstrates good computational efficiency and stability, making it a strong candidate for real-time applications such as video surveillance in coal mines. Furthermore, comparative experiments with other algorithms further confirm the relative advantages and application potential of the algorithm in this paper.

## Conclusion and future work

In response to the urgent need for image dehazing in industrial environments such as coal mines, this paper presents an image dehazing algorithm based on threshold multi-channel prior-based defogging algorithm for underground coal mine images. The algorithm employs an improved color attenuation prior method for fog density detection, enhanced by incorporating texture information in the HSV space and illumination invariance characteristics, thereby improving the algorithm’s adaptability and robustness across varying lighting conditions. During the dehazing process, the algorithm enhances fog-free areas and applies threshold multi-channel inspection to foggy regions, effectively improving the accuracy and reliability of the dehazing results. Furthermore, by constructing a multi-scale pyramid and utilizing a guided filtering approach, the algorithm enables a more refined estimation of image transmittance, alleviating the blocky artifacts common in traditional methods while preserving image details. For video dehazing, a parameter reuse mechanism based on inter-frame similarity is designed, employing perceptual hashing to reduce redundant computations and enhance the real-time performance of video dehazing. In summary, the proposed algorithm is not only applicable to complex industrial settings but also provides new insights and methods for the further development of image dehazing technology.

Although the proposed image dehazing algorithm demonstrates considerable improvements, there remains scope for further enhancement. Specifically, optimizing the algorithm’s performance under extremely low visibility conditions could be achieved by refining the fog density detection models to ensure effective operation in environments with nearly zero visibility. Moreover, the algorithm could benefit from enhancements in processing dynamic scenes, which would entail the development of dynamic scene recognition and adaptive parameter adjustment techniques. Additionally, employing advanced color correction methods could significantly improve color fidelity and accuracy, thereby enhancing the visual quality of dehazed images. Such improvements would not only extend the algorithm’s applicability beyond industrial settings but also enable its generalization across a variety of environments, including urban and natural landscapes.
